# A Treatise on Venereal Diseases

**Published:** 1854-08

**Authors:** 


ART. VI.—*A Treatise on Venereal Diseases.* By A. VIDAL (de Cassis) Surgeon of the Venereal Hospital of Paris. With colored plates. Translated and edited by GEORGE C. BLACKMAN, M. D., Fellow of the Royal Medical and Chirurgical Society of London, etc. New York; Samuel S. & Wm. Wood. 1854.

M. Vidal is the associate of M. Ricord in the Hopital du Midi. Although thus thrown into close contact with that great syphilographer, the doctrines of the two do not harmonize, and they are often found in argumentative collision.

M. Ricord knows but one syphilis—the chancre, and its consecutive accidents. With him blenorrhagia is but a simple inflammation; non-specific, and unattended by consecutive disease. Blenorrhagia is therefore not syphilis. He also affirms that chancre is the only contagion; that secondary and tertiary spmptoms are not communicable by contact, or inoculation. Another of M. Ricord's doctrines is that of "syphilization;" a condition of the system in which it becomes saturated with the poison, and protected from further contagion.

To all of these doctrines M. Vidal opposes a negative. He asserts that, though there may be but one syphilis, there are many contagions; that blenorrhagia is a specific syphilitic poison, unattended by chancre, but capable of producing consecutive disease; that secondary symptoms are in various ways communicable; and, finally, that no such thing as protection by syphilization exists.

Now it seems to us, (with all due deference to the great name of Ricord,) that M. Vidal has the strength of the argument upon his side. In reading Ricord's works, particularly his "Letters on Syphilis," one cannot fail to be struck with the zeal and enthusiasm with which he defends his peculiar dogmas; and especially with the ingenuity with which he twists the most contrary facts into unwilling supports of his theory.

We think that we recognize this difference between the two men: Ricord is a partisan, while Vidal is a calm reasoner.

The present treatise is a large and handsome octavo, very neatly printed, and materially improved by the occasional notes of the editor. Dr. Blackman has done service to the profession in translating this excellent work, and his additions to it, (on the subjects of urinary infiltrations and abscesses) though not strictly within the scope of a work on syphilis, are nevertheless valuable in any place.

For sale by CHAS. J. LEONARD. H.

